# Prognostic prediction in patients with hip fracture: risk factors predicting difficulties with discharge to own home

**DOI:** 10.1007/s10195-011-0138-y

**Published:** 2011-05-04

**Authors:** Tetsuo Hagino, Satoshi Ochiai, Eiichi Sato, Yoshiyuki Watanabe, Shinya Senga, Hirotaka Haro

**Affiliations:** 1 Department of Orthopaedic Surgery, Kofu National Hospital, 11-35 Tenjin-cho, Kofu, Yamanashi, 400-8533 Japan; 2 Department of Orthopaedic Surgery, Faculty of Medicine, University of Yamanashi, Yamanashi, Japan

**Keywords:** Hip fracture, Elderly, Prognosis

## Abstract

**Background:**

Little is known about risk factors that may prevent hip fracture patients from being discharged to home. The present study was developed to investigate possible prognostic factors.

**Materials and methods:**

We studied 345 patients with hip fracture treated at our hospital since 1997, who were living at home before the injury. There were 84 males and 261 females. Mean age at injury was 81.6 years. Fracture type was femoral neck fracture in 152 patients and trochanteric fracture in 193. Patients were divided into those who were discharged to home (home discharge group) and those who were discharged to rehabilitation facilities or died in hospital (non-home discharge group). Gender, age at admission, fracture type, and other factors were investigated. Multivariate analysis was conducted on these variables for the home discharge and non-home discharge groups.

**Results:**

There were 202 patients (58.6%) in the home discharge group and 143 patients (41.4%) in the non-home discharge group. The factors significantly associated with not achieving the goal of discharge to home were age 85 years or above [odds ratio (OR) = 1.79, *P* = 0.0204], chronic systemic diseases (OR = 1.77, *p* = 0.0225), dementia (OR = 3.17, *P* < 0.0001), and walking disability before injury (OR = 5.70, *P* = 0.0328).

**Conclusions:**

In elderly patients with hip fracture, the risk factors that predict difficulties with discharge to home include age at admission, concomitant chronic systemic diseases and dementia, and walking disability before injury.

## Introduction

The ultimate goal of patients who sustain hip fracture is to regain walking ability and return to the patient’s familiar community environment, in other words, his/her own home. This goal is important not only for the patient and medical care providers, but also from the medical economic point of view. However, after hip fracture, patients often have reduced walking ability compared with before injury, and some may even become bedridden or die [1–3, 5, 6, 11]. Thus, many patients do not achieve the goal of discharge to home. In the present study, we reviewed patients treated for hip fracture to examine whether it is possible to predict, at time of admission, those who would have difficulties with discharge to home, and the risk factors involved.

## Materials and methods

Five hundred seventeen patients who sustained hip fracture were admitted to our hospital between 1 January 1997 and 31 December 2008. This hospital is a self-contained regional hospital with an Orthopaedic Department, to care for patients with acute injury, and also an in-hospital rehabilitation facility. However, we do not have affiliated rehabilitation facilities or long-term care facilities. The inclusion criteria in this study were patients who were living in their own home at time of injury and who had femoral neck or trochanteric hip fracture of nonpathologic origin. A total of 345 patients who satisfied these criteria were recruited. The study was approved by the Ethical Committee of our hospital. The study was performed in accordance with the ethical standards of the 1964 Declaration of Helsinki as revised in 2008.

All patients were examined at time of admission and were followed. Information regarding preinjury living status was obtained by interview with the patient or family member. Data on health status and discharge status were collected by reviewing clinical charts during hospitalization and at discharge. The patient population comprised 84 males and 261 females aged from 60 to 103 years (mean 81.6 years) at time of injury. Fracture type was femoral neck fracture in 152 patients and trochanteric fracture in 193 patients. Of 345 patients, 316 received surgery, including hemiarthroplasty in 73 patients and osteosynthesis in 243 patients. The fixation device used was compression hip screw in 103 patients, Hansson pin in 53, gamma nail in 34, cannulated cancellous hip screw in 10, and Ender nail in 1. The remaining 29 patients received conservative treatment, because the patient or family declined surgery or the patient had severe dementia or systemic comorbidity. In this study, the discharge policy was to discharge patients when they had recovered the same level of ambulation as before injury or when their walking ability had reached a plateau.

The patients were divided into those who achieved the goal of discharge to their own home (home discharge group) and those who were not able to return home because of in-hospital death or transfer to rehabilitation hospitals or facilities (non-home discharge group).

The predictor variables examined in this study were gender, patient age, fracture type, anemia, liver function, renal function, electrolyte abnormality, urinary glucose, inflammatory status, lung function, cardiac function, chronic systemic comorbidity, cognitive level at admission, and walking ability before injury (can walk unaided or aided by a cane) (Table 1). These predictor variables have been described in previous work from our institution [3].

**Table 1:** Evaluation items at admission

1.	Gender	Male
2.	Age	85 years or above
3.	Fracture type	Femoral neck fracture
4.	Anemia	Hemoglobin 12 g/dl or lower for men, 11 g/dl or lower for women
5.	Liver function	GOT 40 1U/I or above, GPT 35 IU/I or above
6.	Renal function	BUN 20 mg/dl or above
7.	Electrolyte abnormality	Positive
8.	Urinary glucose	Positive
9.	Inflammation status	CRP 0.5 mg/dl or above
10.	Lung function	Abnormal chest X-ray and with a medical diagnose
11.	Heart function	Abnormal ECG (arrhythmia, ischemic changes, etc.)
12.	Chronic systemic disease^a^	Presence or absence of diabetes, congestive heart failure, ischemic heart disease, etc.
13.	Dementia	Present
14.	Walking ability before injury	Can walk unaided or aided by cane

^a^Eleven diseases as described previously, *GOT* glutamate oxaloacetate transaminase, *GPT* glutamate pyruvate transaminase, *BUN* blood urea nitrogen, *ECG* electrocardiogram, *CRP* C-reactive protein

Univariate analysis for each of these items was conducted comparing the home discharge and non-home discharge groups. The factors that showed significant differences (*P* < 0.05) on univariate analysis were included as independent variables in the subsequent multivariate analysis. Multivariate analysis was performed by logistic regression using the above-mentioned independent variables and discharge to home as the dependent variable.

Furthermore, patients were divided by treatment modality: 73 treated by hemiarthroplasty, 243 by osteosynthesis, and 29 by conservative methods, and the rates of home discharge and non-home discharge in each group were tabulated. The relation between treatment modality and home discharge was analyzed by chi-square test for independence.

All statistical analyses were performed using StatView 5.0 statistics software. *P* value less than 0.05 was considered significant.

## Results

There were 202 patients (58.6%) in the home discharge group and 143 patients (41.4%) in the non-home discharge group. Seven patients in the non-home discharge group died during hospitalization. Length of hospital stay was 76.7 ± 35.3 days in the home discharge group and 58.9 ± 37.7 days in the non-home discharge group.

Univariate analyses revealed significant differences for seven variables: age, renal function, electrolytes, lung function, chronic systemic diseases, dementia, and walking ability before injury (Table 2). No significant differences were observed for gender, fracture type, anemia, liver function, lung function, glucosuria, inflammatory finding, and ECG. Multivariate analysis identified the following four factors to be significantly associated with not achieving the goal of discharge to home: age 85 years or above [odds ratio (OR) = 1.79, *P* = 0.0204], chronic systemic diseases (OR = 1.77, *P* = 0.0225), dementia (OR = 3.17, *P* < 0.0001), and walking disability before injury (OR = 5.70, *P* = 0.0328) (Table 3).

**Table 2:** Univariate analyses of factors associated with difficulties with discharge to home

	Home discharge group (*n* = 202) (*n*%)	Non-home discharge group (*n* = 143) (*n*%)	*P* value group
Male	47 (23.3)	37 (25.9)	N.S.
Age (85 years or above)	65 (32.2)	74 (51.7)	*P* < 0.0005
Femoral neck fracture	93 (46.0)	59 (41.3)	N.S.
Anemia	83 (41.1)	67 (46.9)	N.S.
Abnormal liver function	14 (6.9)	15 (10.5)	N.S.
Abnormal renal function	68 (33.7)	70 (49.0)	*P* < 0.005
Abnormal electrolytes	45 (22.3)	48 (33.6)	*P* < 0.00001
Glucose in urine positive	34 (16.8)	23 (16.1)	N.S.
Inflammatory finding present	125 (61.9)	86 (60.1)	N.S.
Abnormal lung function	11 (5.4)	20 (14.0)	*P* < 0.01
Abnormal ECG	53 (26.2)	50 (35.0)	N.S.
Chronic systemic diseases	105 (52.0)	99 (69.2)	*P* < 0.005
Dementia present	28 (13.9)	57 (39.9)	*P* < 0.00001
Ambulatory before injury	200 (99.0)	133 (93.0)	*P* < 0.005

Analyzed by chi-square for independence teat, or Fisher’s exact probability test, *N.S.* not significant

**Table 3:** Risk factors predicting difficulties with discharge to home

Risk factors	Odds ratio (95% confidence interval)	*P* value*
Age	1.79 (1.09–2.93)	0.0204
Chronic systemic diseases	1.77 (1.08–2.90)	0.0225
Dementia	3.17 (1.83–5.52)	<0.0001
Walking disability before injury	5.70 (1.15–28.19)	0.0328

* Logistic regression analysis

Chi-square test for independence showed no relation between treatment modality and difficulties with home discharge (Table 4).

**Table 4:** Relation between treatment modality and difficulties with discharge to home

	Home discharge group (*n* = 202)	Non-home discharge group (*n* = 143)	Total
Hemiarthroplasty*	47	26	73
Osteosynthesis*	142	101	243
Conservative treatments*	13	16	29

* No significant difference by chi-square test for independence (*P* = 0.194)

## Discussion

Osnes et al. [8] reported that the proportion of patients with hip fracture living in nursing homes increased from 15% before fracture to 30% after the injury. Furthermore, Holt et al. [4] found that, among patients who were living at home before sustaining fracture injury, only 51% of the extremely elderly patients were living at home at 120 days after injury, and they concluded that the extremely elderly patients were less likely to return home. In the present study of 345 patients who were living at home before injury, only 202 patients (58.6%) were discharged to their own home. Despite a relatively long hospital stay, many of the patients were not able to return home but were transferred to rehabilitation hospitals or facilities. Tsuboi et al. [11] followed 753 patients for 10 years after hip fracture and reported that the proportion of patients living at home was 84% before fracture, decreasing to 60% at 120 days after fracture but improving to 81% at 1 year, and then remaining stable at approximately 86% until 10 years later. Among our patients who were discharged to rehabilitation hospitals or facilities, some would have returned home after completion of rehabilitation. However, since follow-up was not possible after the transfer, the details remain unknown. A follow-up investigation should be conducted in the future.

A few studies have examined the risk factors predicting difficulties with discharge to home. Samuelsson et al. [9] studied 2,134 patients with hip fracture and found that cognitive function was the most important factor for returning to own home and regaining prefracture function. Nori et al. [7] investigated 123 elderly patients with hip fracture and identified daytime nursing care and dementia as independent factors. Thorngren et al. [10] reported that the most important favorable variables for discharge to home were (1) ability to walk 2 weeks after surgery, (2) living with someone, and (3) good general health, while the negative variable was old age. Our analysis identified age at admission, concurrent chronic systemic diseases and dementia, and walking disability before injury as risk factors predicting difficulties with discharge to home. Since most of these risk factors already exist at time of admission, amelioration of these factors by interventions from the medical team is probably unlikely. However, to increase the rate of returning home, hospital discharge planning should be started from the early stage of hospitalization with cooperation from the family or care providers, to prepare a supportive environment and outline rehabilitation and nursing care plans after discharge. In this regard, early prediction of prognosis is important.
